# Pro-Inflammatory and B Cell Regulating Capacities of TWEAK in Rainbow Trout (*Oncorhynchus mykiss*)

**DOI:** 10.3389/fimmu.2021.748836

**Published:** 2021-10-01

**Authors:** Beatriz Abós, Elena Pérez-Fernández, Esther Morel, Pedro Perdiguero, Carolina Tafalla

**Affiliations:** Animal Health Research Center (CISA), Centro Nacional Instituto de Investigación y Tecnología Agraria y Alimentaria (INIA), Consejo Superior de Investigaciones Científicas (CSIC), Madrid, Spain

**Keywords:** tumor necrosis factor superfamily (TNFSF), TNF-like weak inducer of apoptosis (TWEAK), rainbow trout, head kidney, B cells, inflammation

## Abstract

Tumor necrosis factor (TNF)-like weak inducer of apoptosis or TWEAK is a member of the TNF superfamily involved in the regulation of many biological processes. In mammals, TWEAK has been shown to play a role in some autoimmune or inflammatory conditions, but its immune role is not yet clearly defined. In teleost fish, although a few studies have identified homologues to mammalian TWEAK, their biological effects have never been investigated. In the current study, we have studied the transcriptional regulation of two TWEAK homologues (TWEAK 1 and 2) identified in rainbow trout (*Oncorhynchus mykiss*) throughout different tissues, in response to parasitic or viral infections, or in head kidney (HK) leukocytes stimulated with different stimuli. Although the transcription of both homologues was modulated when HK leukocytes were exposed to several immune stimuli, only TWEAK 1 was significantly modulated upon pathogenic exposure. Thus, we performed a characterization of the functions exerted by this cytokine in HK leukocytes. Recombinant TWEAK 1 strongly up-regulated the transcription of pro-inflammatory genes and antimicrobial peptides in HK leukocytes, with differential transcriptional effects in IgM^+^ B cells, IgM^-^ lymphocytes and myeloid cells. TWEAK 1 also increased the survival and promoted the differentiation of B cells in HK leukocyte cultures. Our results demonstrate that in teleost fish, TWEAK 1 is involved in the response to different types of pathogens, through the modulation of antimicrobial and pro-inflammatory genes in different leukocytes subsets. Furthermore, a role for TWEAK as a B cell differentiation factor has also been established in rainbow trout.

## Introduction

In mammals, the tumor necrosis factor superfamily (TNFSF) of cytokines activates signaling pathways that are involved in different cellular activities such as organogenesis, survival, apoptosis, inflammation, lymphocyte homeostasis, or cellular differentiation (1). There are 19 known TNFSF ligands described in human and mouse (2). These ligands are type II membrane-bound proteins with an intracellular N terminal and an extracellular C terminal domain. The TNF-like weak inducer of apoptosis (TWEAK) is one of these ligands (TNFSF12) and can be found as a homotrimeric type II transmembrane protein or as a soluble protein released by furin proteases (3). Both forms can be expressed simultaneously but the mechanisms that control their relative production are still not known. TWEAK mediates several cellular and inflammatory responses by binding to a TNF receptor known as fibroblast growth factor-inducible molecule 14 (Fn14). Even though some studies have pointed to an interaction of TWEAK with other receptors such as CD163 (scavenger receptor expressed on monocytes/macrophages) (4), at the time, Fn14 is the only confirmed competent receptor for TWEAK and TWEAK the only ligand for Fn14. TWEAK and Fn14 genes are expressed at low levels in most normal tissues but are up-regulated in damaged tissues (5, 6). Interestingly, the TWEAK-Fn14 pathway has recently been associated with the pathogenesis of several autoimmune disorders including rheumatoid arthritis (RA), systemic lupus erythematosus (SLE) and multiple sclerosis (MS) (7, 8).

Leukocytes seem to be a major source of TWEAK, with TWEAK mRNA widely detected throughout the human hematopoietic lineage, including monocytes, dendritic cells (DCs), neutrophils, natural killer (NK) cells and T cells (3, 9–11). This constitutive transcription has been shown to increase during inflammation or stimulation (3). Thus, for example, up-regulation of TWEAK transcription was reported in human monocytes treated with interferon γ (IFN-γ) (12). LPS, on the other hand, was able to increase TWEAK mRNA levels in human THP-1 monocytic cells (13), whereas it down-regulated them in mice peritoneal macrophages (14). Additionally, TWEAK expression is induced on the cell surface of human NK cells, monocytes and DCs in response to IFN-γ and PMA stimulation (11). Membrane TWEAK has also been detected in freshly isolated monocytes from MS patients (15) and on T cells from patients with SLE (9). Interestingly, TWEAK expression has also been reported in many non-hematopoietic tissues, such as heart, kidney, lung or placenta (16, 17), as well as in tumor samples (18–21) and tumoral cell lines (3, 18, 20, 21). On the other hand, Fn14 is highly inducible in epithelial cells, endothelial cells and other mesenchymal cell types in response to several growth factors and pro-inflammatory cytokines (5), or to CpG DNA (22). Fn14 is also constitutively expressed in macrophages, NK cells and DCs, being up-regulated by stimulation with IFN-γ or PMA (11).

In mammals, to date, the effects of TWEAK on adaptive immune responses have not been well defined. A study performed in a murine model of SLE, revealed that when splenic B cells from these animals were treated with recombinant TWEAK for 3 days, a significant up-regulation of the levels of transcription of several genes involved in B cell maturation and germinal center (GC) formation such as activation-induced cytidine deaminase (AID), B lymphocyte-induced maturation protein 1 (Blimp1) or interferon regulatory factor 4 (IRF4) was observed (23). In these mice, the inhibition of the TWEAK/Fn14 signaling pathway significantly suppressed Ig production, GC formation and B cell differentiation with reduced mRNA levels of X-box binding protein 1 (Xbp-1), Blimp1 and IRF4 (23). This was accompanied by a reduction of the T follicular helper cell population. Similarly, the presence of a human autosomal dominant TWEAK mutation induced a decrease in IgM and IgA levels together with a reduction in B cell survival and proliferation, pointing to an important role of TWEAK in B cell functionality (24). Despite this, a full understanding of how TWEAK regulates B cell functionality in mammals is still lacking.

In teleost fish, TWEAK homologues have been identified in some species such as zebrafish (*Danio rerio*), grass carp (*Ctenopharyngodon idella*), fugu (*Fugu rubripes*) tetraodon (*Tetraodon nigroviridis*) and cartilaginous fish such as the Australian ghost shark (*Callorhinchus milii*) and the whale shark (*Rhincodon typus*) (25–28). Surprisingly, TWEAK is known to be absent in the genome of other species, suggesting a non-essential function in some fish (28). In both zebrafish and grass carp, higher constitutive levels of TWEAK transcription were observed in spleen, kidney and gills as well as in the skin in the case of the grass carp (27). Besides, TWEAK transcription was up-regulated in response to *Aeromonas hydrophila* or *Aquareovirus* infection in several grass carp organs (27). However, to date, no studies have investigated the functional effects of teleost TWEAK. Interestingly, TWEAK, as well as two homologues of the TWEAK receptor, were among the genes that were significantly modulated in rainbow trout (*Oncorhynchus mykiss*) splenic IgM^+^ B cells in response to stimulation with either TNP-LPS or TNP-KLH (29). This led us to investigate further the role of TWEAK in the rainbow trout immune response. Thus, in the current work, we studied the transcriptional regulation of two TWEAK homologues (designated as TWEAK 1 and 2) identified in rainbow trout and we performed a functional analysis after recombinantly producing TWEAK 1. We evaluated the constitutive levels of transcription of TWEAK 1 and 2 in different fish tissues and head kidney (HK) leukocyte subpopulations, given that in teleost fish the HK is the main hematopoietic tissue and the site for B cell maturation in the absence of bone marrow (30). We then studied the transcription of TWEAK 1 and 2 in response to different pathogenic experiences, that is fish affected by proliferative kidney disease (PKD), a disease caused by the myxozoan parasite *Tetracapsuloides bryosalmonae* and fish infected with viral hemorrhagic septicemia virus (VHSV). Finally, we determined how TWEAK transcription was modulated in stimulated HK leukocytes. Our results revealed that although both homologues were transcriptionally regulated in HK leukocytes in response to different stimuli, only TWEAK 1 was significantly affected in PKD or VHSV infection. Thus, we focused on this homologue to study the biological activity of TWEAK in rainbow trout. Recombinant rainbow trout TWEAK 1 cytokine had the capacity to increase the transcription of pro-inflammatory genes and antimicrobial peptides, with different effects on sorted leukocyte subpopulations (IgM^+^ B cells, IgM^-^ lymphocytes and IgM^-^ myeloid cells). To provide further insights on the B cell regulating role of TWEAK, we also studied trout B cell responses to the recombinant cytokine. Hence, TWEAK 1 increased IgM^+^ B cell survival and induced B cell differentiation increasing the number of IgM^+^ secreting cells and up-regulating the transcription of genes encoding homologues of Blimp1 in IgM^+^ sorted B cells. In conclusion, our results provide insights on the regulation and functionality of teleost TWEAK, a member of the TNFSF not widely investigated in these species.

## Material and Methods

### Experimental Fish

Rainbow trout (*Oncorhynchus mykiss*) of approximately 100 g obtained from *Piscifactoría Cifuentes* (Guadalajara, Spain) were used to study the constitutive levels of TWEAK transcription and to isolate head kidney leukocytes. These fish were maintained at the animal facilities of the Animal Health Research Center (CISA-INIA/CSIC) in a recirculating water system at 14°C, with a 12:12-h light/dark photoperiod. Fish were fed a commercial diet (Skretting) twice a day. Trout were acclimatized to laboratory conditions for at least 2 weeks before any experimental procedure.

### RNA Extraction From Tissues Obtained From Naïve Rainbow Trout

Fish were anaesthetized with 50 mg/ml benzocaine (Sigma) following the recommendations from Zahl et al. (31). Blood was extracted with a heparinized needle from the caudal vein. Prior to sampling, a transcardial perfusion was conducted to remove all circulating blood from tissues. For this, the heart was cannulated through the ventricle into the bulbus arteriosus with approximately 30 ml of 0.9% NaCl, using a peristaltic pump (Selecta, Spain), while the atrium was cut to drain the blood out of the circulatory system. After perfusion, different tissues (spleen, HK, posterior kidney, muscle, heart, gonad, skin, gill, liver, brain, thymus, esophagus, stomach, pyloric caeca, midgut, hindgut and adipose tissue) were collected and placed in TRIzol (Invitrogen) for subsequent RNA isolation.

Total RNA was extracted from tissue samples using a combination of TRIzol and RNAeasy Mini kit (Qiagen) previously described (32). Briefly, samples were mechanically disrupted in 1 ml of TRIzol using a disruption pestle. Then, 200 µl of chloroform were added and the suspension was centrifuged at 12,000 x *g* for 15 min. The clear upper phase was recovered, mixed with an equal volume of 100% ethanol and immediately transferred to RNAeasy Mini kit columns. The procedure was then continued following manufacturer’s instructions, performing on-column DNase treatment. Finally, RNA was eluted from the columns in RNase-free water, quantified in a Nanodrop 1000 spectrophotometer (Thermo Scientific) and stored at -80°C until use. One µg of total RNA for each tissue was used to synthesize cDNA using the RevertAid Reverse Transcriptase (Thermo Scientific), primed with oligo(dT)_23_VN (1.6 µM), following the manufacturer´s instructions. The resulting cDNA was diluted in a 1:5 proportion with water and stored at -20°C.

### *In Silico* Identification and Analysis of Rainbow Trout TWEAK Homologues

Rainbow trout genes homologues to TWEAK were identified by comparative analysis using the Blastp software. The TWEAK protein previously characterized in zebrafish (ADM64322.1; 26) was used as a query against all proteins identified in the rainbow trout genome (available in the Refseq protein database). In order to analyze the evolution of TWEAK, several proteins annotated as “tumor necrosis factor ligand superfamily member 12” or “12-like” were retrieved from the Refseq protein database covering different classes (mammals, frogs, reptiles, amphibians and fish). A set of filtered proteins was included in a multiple protein alignment using the ClustalW software. A phylogenetic tree was constructed using the maximum likelihood algorithm in the MEGA X software and the analysis of reliability assessed by 1000 bootstrap replicates. A protein homologue to TNFSF12 identified in lancelet *Branchiostoma floridae* was included as an outgroup and used for rooting purposes.

### Analysis of Transcription of TWEAK Homologues

To evaluate the levels of transcription of the two TWEAK homologues, real-time PCR was performed in a LightCycler 96 System instrument (Roche) using FastStart Essential DNA Green Master reagents (Roche) and specific primers (listed in **Supplementary Table S1**). The efficiency of the amplification was determined for each primer pair using serial 10-fold dilutions of pooled cDNA, and only primer pairs with efficiencies between 1.95 and 2 were used. Each sample was measured in duplicate under the following conditions: 10 min at 95°C followed by 40 amplification cycles (30 s at 95°C and 1 min at 60°C). The expression of individual genes was normalized to relative expression of trout EF-1α, and the expression levels calculated using the 2^−ΔCt^ method, in which Δ threshold cycle (Ct) is determined by subtracting the EF-1α value from the target Ct. A melting curve for each PCR was determined by reading fluorescence every degree between 60 and 95°C to ensure only a single product had been amplified. Negative controls with no template were included in all the experiments.

### VHSV Infection and Sampling

Samples from rainbow trout infected with viral hemorrhagic septicemia virus (VHSV) were obtained from an experiment previously described (33). The VHSV strain used was 0771, propagated in the RTG-2 rainbow trout cell line (34). For the infection, rainbow trout of approximately 10 g were divided in two groups of 20 trout each. Groups were injected intraperitoneally with either 100 µl of culture medium (mock-infected control) or 100 µl of a viral solution containing 1 x 10^6^ TCID_50_/ml (tissue culture infective dose per ml). At days 1, 3, and 7 post-injection, six trout from each group were sacrificed by overexposure to benzocaine, and HK and spleen sampled and placed in TRIzol. RNA was isolated following the manufacturer´s instructions and cDNA obtained as described above for tissues obtained from naïve fish. The levels of transcription of the different TWEAK homologues were evaluated through real time PCR as described above.

### PKD Infected Fish Sampling

Samples from rainbow trout naturally exposed to *Tetracapsuloides bryosalmonae* were obtained from an experiment previously described (35). Briefly, kidney samples were classified in different swelling grades (1–3) attending to the clinical stage of PKD as described by Clifton-Hadley et al. (36). Kidneys were then placed into 1 ml of RNA-later (Sigma), kept at 4°C for 24 h and stored at -80°C until use. For RNA extraction, kidney samples were homogenized in 1.5 ml of TRI Reagent (Invitrogen) and tissue debris removed by centrifugation (12,500 x *g* for 10 min at 4°C). Total RNA was isolated following the manufacturer´s instructions and diluted in TE buffer (pH 8.0) at -80°C until use. cDNA was then produced and TWEAK 1 and 2 gene transcription levels assessed as described above.

### Production of Recombinant Rainbow Trout TWEAK 1

The protein sequence corresponding to TWEAK 1 (XP_021433661.1) was analyzed using the PSIPRED workbench (http://bioinf.cs.ucl.ac.uk/psipred/) in order to identify the different features of the amino acid sequence (**Supplementary Figure S1A**). The nucleotide sequence corresponding to the extracellular region of the protein was synthetized and subcloned into the E3 expression vector (Abyntek) together with an N-terminal 6x histidine tag (**Supplementary Figure S1B**). The signal peptide and sequences that potentially interact with the plasma membrane were discarded keeping exclusively amino acid sequence corresponding to the extracellular region of rainbow trout TWEAK 1. The recombinant plasmid was transformed into BL21 cells and a kanamycin-resistant single positive colony was then incubated at 37°C in Luria-Bertani (LB) media. When the OD600 reached 0.6, 0.1 mM of isopropyl β-D-thiogalactoside (IPTG, Sigma Aldrich) was added to induce protein production. After 16 h, cells were harvested, lysed by sonication and dissolved using urea. Thereafter, TWEAK 1 was obtained through the use of Nickel columns (Sigma Aldrich). The TWEAK 1-containing fractions were pooled, refolded, filtered through 0.22 μm and resuspended in storage buffer (50 mM Tris-HCl, 150 mM NaCl, 10% glycerol, 0.5 M L-arginine, pH 8). Protein concentration was determined in a BCA protein assay (Thermo Fisher Scientific) and the recombinant rainbow trout TWEAK 1 (0.3 mg/ml) was aliquoted and stored at -80°C until used. The levels of endotoxin in the protein were determined with a LAL Endotoxin Assay kit (Thermo Fisher Scientific). An irrelevant protein (in this case lymphotoxin β1) with a similar molecular weight to that of recombinant TWEAK 1 (22 KDa), also bearing an N-terminal His tag, was produced in the same conditions (C-His), and used as a functional control. This protein contained similar endotoxin levels as TWEAK 1.

### Head Kidney Leukocyte Isolation

Fish were sacrificed by benzocaine overdose. HKs were collected and single cell suspensions obtained using 100 μm nylon cell strainers (BD Biosciences). Cells were then diluted in Leibovitz’s medium (L-15, Invitrogen) supplemented with 100 IU/ml penicillin and 100 μg/ml streptomycin (P/S, Life Technologies), 2% fetal calf serum (FCS, Life Technologies) and 10 U/ml heparin (Sigma) and placed onto 30/51% discontinuous Percoll density gradients for centrifugation at 500 x *g* for 30 min at 4°C, without brake. Cells at the interface were collected and washed in L-15 containing antibiotics and 2% FCS. The viable cell concentration was determined by Trypan blue (Sigma-Aldrich) exclusion using a Neubauer chamber.

### TWEAK 1 and 2 Regulation in HK Leukocytes

To study how different stimuli could influence the levels of transcription of TWEAK 1 and 2, HK leukocytes adjusted to 2 x 10^6^ cells/ml in L-15 containing antibiotics and 5% FCS were seeded in 24-well plates and then stimulated with either PMA (100 ng/ml), LPS (100 µg/ml), Poly I:C (50 µg/ml), recombinant rainbow trout IFN-γ (25 ng/ml) or recombinant rainbow trout IL-1β (20 ng/ml). PMA, LPS and Poly I:C were all obtained from Sigma, whereas recombinant cytokines were provided by Dr. Tiehui Wang and Prof. Chris Secombes (University of Aberdeen, UK). Cells with media alone were included as controls. After 24 h of incubation at 20°C, RNA was extracted from the cells with TRI Reagent following the manufacturer´s instructions and the levels of transcription of TWEAK 1 and 2 determined as described above.

### Transcriptional Effects Provoked by TWEAK 1 in HK Leukocytes

In other experiments, HK leukocytes adjusted to a concentration of 2 x 10^6^ cells/ml in L-15 medium supplemented with antibiotics and 5% FCS, were seeded in 24-well plates and incubated at 20°C in the presence of 0.01, 0.1 and 1 µg/ml of recombinant TWEAK 1. Doses of 1 µg/ml have been previously used in mammals in *in vitro* studies involving recombinant TWEAK (37, 38). Non-stimulated controls were always included as well as controls exposed to the same volume of TWEAK 1 storage buffer. To determine the transcriptional effects of TWEAK 1, cells were collected in TRI Reagent after 24 h of incubation with the different TWEAK 1 concentrations. RNA was isolated following the manufacturer´s instructions, cDNA produced as described above and the levels of transcription of several immune genes determined using primers and protocols previously optimized (**Supplementary Table S1**) (39–41). Each sample was measured in duplicate under the following conditions: 10 min at 95°C followed by 40 amplification cycles (30 s at 95°C and 1 min at 60°C). A melting curve for each PCR was determined by reading fluorescence every degree between 60 and 95°C to ensure only a single product had been amplified in each case. Negative controls with no template were included in all the experiments. The expression of individual genes was normalized to the relative expression of the housekeeping gene EF-1α and calculated as described above.

### Cell Sorting

IgM^+^ B cells, IgM^-^ lymphoid cells (small cells with low complexity) and IgM^-^myeloid cells (large cells with high complexity) were sorted from HK leukocytes cultures by flow cytometry using a BD FACSAria III cell sorter (BD Biosciences). For this purpose, HK leukocytes were seeded in 24-well plates at a density of 2 x 10^6^ cells per ml and incubated for 24 h at 20°C with 1 µg/ml of TWEAK 1 or media alone. Thereafter, cells were collected and incubated for 20 min at 4°C in the dark with an anti-IgM (1.14 mAb mouse IgG_1_ coupled to FITC, 2 µg/ml) (42) in staining buffer (phenol red-free L-15 medium supplemented with 100 IU/ml penicillin and 100 μg/ml streptomycin and 2% FCS). Following two washing steps, cells were resuspended in staining buffer and counterstained with 7-aminoactinomycin D (7-AAD, BD Biosciences, 2.5 µg/ml) to exclude dead cells. Next, IgM^+^ B cells, IgM^-^ lymphoid cells and IgM^-^ myeloid cells were isolated based on their FSC/SSC profiles and on the fluorescence emitted by the anti-IgM Ab conjugated to FITC (**Supplementary Figure S2**). Approximately 50,000 cells of each population were sorted from stimulated and control HK leukocytes for subsequent RNA isolation.

### RNA Extraction and Real Time PCR Analysis of Sorted Leukocyte Subsets

Total RNA was isolated from FACS isolated cell populations using the Power SYBR Green Cells-to-Ct Kit (Invitrogen) following the manufacturer’s instructions. RNA was treated with DNase during the process to remove genomic DNA that might interfere with the PCR reactions. Reverse transcription was also performed using the Power SYBR Green Cells-to-Ct Kit following the manufacturer’s instructions. To evaluate the levels of transcription of the different genes, real time PCR was performed with a LightCycler R 96 System instrument using SYBR Green PCR core Reagents (Applied Biosystems) and specific primers (**Supplementary Table S1**). Samples obtained from individual fish were analyzed in duplicate under the following conditions: 10 min at 95°C, followed by 40 amplification cycles (15 s at 95°C and 1 min at 60°C). A melting curve for each PCR was determined by reading fluorescence every degree between 60 and 95°C to ensure only a single product had been amplified in each case. Negative controls with no template were included in all the experiments. The expression of individual genes was normalized to the relative expression of the housekeeping gene EF-1α and calculated as described above.

### Flow Cytometry Analysis

HK leukocytes seeded in 96-well plates (Nunc) at a concentration of 2 x 10^6^ cells/ml, were stimulated with 0.01, 0.1 and 1 µg/ml of TWEAK 1 and cultured at 20°C for 3 days. Non-stimulated controls were also included as well as controls exposed to the same volume of TWEAK 1 storage buffer. After this time, 4 x 10^5^ HK leukocytes were collected, washed in staining buffer and incubated with an anti-trout IgM [1.14 mAb mouse IgG_1_ coupled to phycoerythrin (PE), 1 µg/ml] and an anti-trout IgD [mAb mouse IgG_1_ conjugated to allophycocyanin (APC), 10 µg/ml] (43). Cells were incubated in staining buffer for 1 h at 4°C in the dark in the presence or absence of the Abs. Afterwards, cells were washed twice and analyzed on a FACS Celesta flow cytometer (BD Biosciences) equipped with BD FACSDiva™ software. Dead cells were excluded from the analysis after staining with 4’,6-diamine-2’-phenylindole dihydrochloride (DAPI, 0,2 µg/ml). Flow cytometry analysis was performed with FlowJo^®^ v.10 (FlowJo LLC, Tree Star) following the gating strategy described in **Supplementary Figure S3**.

### ELISpot

ELISpot was used to measure the number of IgM secreting B cells in HK leukocyte cultures. For this, HK leukocytes were seeded in 96-well plates in triplicate at a concentration of 5 x 10^4^ cells per well. Cells were then stimulated with or without 0.01, 0.1 and 1 µg/ml of recombinant TWEAK 1 for 48 h at 20°C. During this time, ELISpot plates containing Immobilon-P membranes (Millipore) were activated with 70% ethanol and coated with 2 µg/ml of an anti-trout IgM mAb (clone 4C10) at 4°C with shaking. Plates were then washed with sterile PBS (phosphate buffer saline) five times and non-specific binding sites were blocked by incubation with 2% BSA in PBS for 2 h at room temperature (RT). After the 48 h incubation period, cells were transferred to ELISpot plates and incubated for a further 24 h. Next, leukocytes were removed from the plates and the plates washed 5 times with PBS and blocked with 2% BSA (bovine serum albumin) in PBS for 1 h at RT. After blocking, biotinylated anti-trout IgM mAb (clone 4C10) was added to the plates (1 µg/ml) and incubated for 1 h at RT. Following additional washing steps (5 times in PBS), the plates were developed using streptavidin-HRP (Thermo Fisher Scientific) at 100 ng/ml for 1 h at RT, washed again with PBS and incubated with 3-amino 9-ethylcarbazole (Sigma-Aldrich) for 30 min at RT in the dark. The substrate reaction was stopped by washing the plates with tap water. Once the membranes were dried, the number of spots in each well was determined using an AID iSpot Reader System (Autoimmun Diagnostika GMBH).

### Statistical Analysis

Data was analyzed using Microsoft Office Excel 2010. Statistical analyses were performed using a two-tailed Student’s *t* test with Welch’s correction when the F test indicated that the variances of both groups differed significantly. To establish differences among the levels of TWEAK transcription in fish affected by PKD at different degrees, a one-way ANOVA was conducted followed by a Dunnett*’s* test. The differences between the mean values were considered significant on different degrees, where * means *p* ≤ 0.05; ** means *p* ≤ 0.01 and *** means *p* ≤ 0.001.

## Results

### Salmonid Genomes Contain Two Genes Encoding TWEAK

The *in silico* identification of rainbow trout homologs highlighted that this species contains two different genes encoding TWEAK homologues located in different chromosomes along the genome. The first gene (LOC110500573), located in Chromosome 21, encodes the protein with Refseq accession XP_021433661.1, whereas the second gene (LOC110531703) is located in Chromosome 9, encoding the protein with Refseq accession XP_036843426.1. The alignment between both proteins showed a high degree of conservation between them, with 88% of amino acid similarity (**Supplementary Figure S4A**). A phylogenetic tree was then constructed, rooted with a TNFSF12-like gene from lancelet (*B. floridae*). Within the tree, from an evolutionary point of view, a first group is formed by TWEAK proteins from chondrichthyes (cartilaginous fish) while the following node includes tetrapod TWEAK proteins as well as that of the extant fish *Latimeria chalumnae* (**Supplementary Figure S4B**). Thus, proteins from human, mouse, amphibians and reptiles are closely related with *L. chalumnae* TNFSF12, suggesting that these proteins in mammals and fish evolved from a common ancestor (**Supplementary Figure S4B**). The phylogenetic analysis highlighted that while some fish such as zebrafish only contain one TWEAK homologue, rainbow trout and other salmonid species contain two gene copies in two different locations along genome, for instance: *Oncorhynchus kisutch*, LOC109906839 in LG16 and LOC109897008 in LG9; *Oncorhynchus keta*, LOC118392010 in LG13 and LOC118368797 in LG35; *Salmo salar*, LOC106608761 in ssa07 and LOC106577651 in ssa18; or *Salvelinus alpinus*, LOC111960055 in LG37 and LOC111953797 in LG3. The proteins encoded by these genes in salmonids form two clearly differentiated groups in the phylogenetic tree, which we designated as TWEAK 1 and TWEAK 2 (**Supplementary Figure S4B**).

### TWEAK 1 and TWEAK 2 Transcription in Trout Tissues and HK Leukocyte Subpopulations

**Figure 1:**
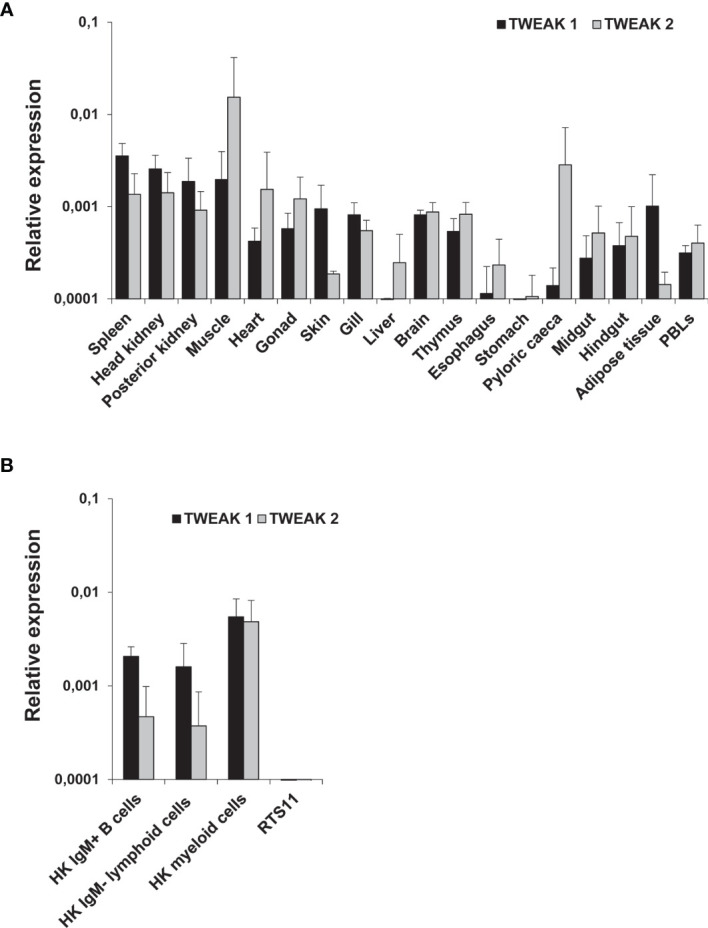
Constitutive levels of TWEAK 1 and 2 transcription in different tissues **(A)** and HK leukocyte subpopulations **(B)** from unstimulated rainbow trout. The levels of TWEAK 1 and 2 transcription were estimated by real-time PCR in different trout tissues (n = 3), as well as in sorted HK leukocyte subpopulations (IgM^+^ B cells, IgM^-^ lymphoid cells and myeloid cells) and the RTS11 cell line (n = 6). Gene expression data was normalized against the endogenous control EF-1α and is shown as relative expression levels (mean + SD).

The constitutive levels of transcription of TWEAK 1 and 2 were evaluated in different tissues from healthy unstimulated rainbow trout. No comparisons were made between TWEAK 1 and TWEAK 2 transcription levels for one tissue, but only between tissues for a single gene. The higher TWEAK 1 expression levels were found in spleen, HK, posterior kidney and muscle (**Figure 1A**). Then again, TWEAK 2 transcriptional levels were highest in muscle, pyloric caeca, heart, HK and spleen (**Figure 1A**). TWEAK 1 and 2 transcription was also analyzed in FACS-isolated leukocyte populations from HK, including IgM^+^ B cells, IgM^-^ lymphoid cells (small cells with low complexity) and IgM^-^ myeloid cells (large cells with high complexity) (**Supplementary Figure S2**). In this case, the highest levels of TWEAK 1 and 2 transcripts were found in HK myeloid cells in comparison to IgM^+^ B cells and IgM^-^ lymphoid cells (**Figure 1B**). Similar transcriptional levels were observed for TWEAK 1 and 2 in HK myeloid cells, whereas in IgM^+^ B cells and IgM^-^ lymphoid cells the levels of transcription of TWEAK 2 were lower than those of TWEAK 1 (**Figure 1B**). Surprisingly, TWEAK 1 and 2 transcription was undetectable in the RTS11 monocyte-macrophage rainbow trout cell line (**Figure 1B**).

### Regulation of TWEAK 1 and 2 Transcription in Response to VHSV Infection

**Figure 2:**
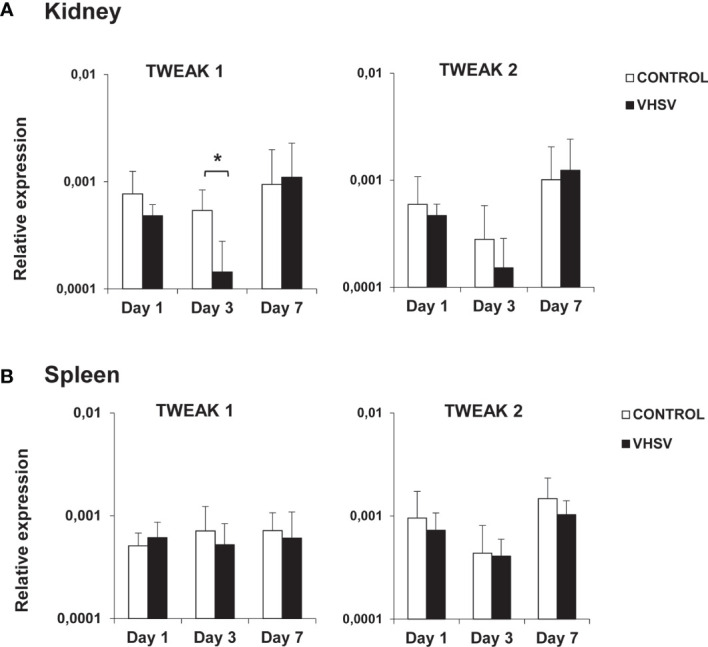
Levels of transcription of TWEAK 1 and 2 in kidney **(A)** and spleen **(B)** of rainbow trout challenged with VHSV by intraperitoneal injection. Trout were intraperitoneally injected with either 100 µl of culture medium (control) or 100 µl of a viral solution containing 1 x 10^6^ TCID_50_/ml VHSV. At days 1, 3 and 7 post-infection, 6 fish in each group were sacrificed and the levels of transcription of TWEAK 1 and 2 in kidney and spleen determined by real-time PCR. Data are shown as the gene expression relative to the expression of an endogenous control EF-1α (mean + SD, n = 6). Asterisks represent statistically significant differences between the values obtained in infected fish and those in mock-infected controls (*p < 0.05).

To establish if TWEAK is implicated in the teleost antiviral response, we studied the levels of transcription of both TWEAK homologues in rainbow trout challenged with VHSV. Interestingly, VHSV provoked a significant down-regulation of TWEAK 1 mRNA levels in the kidney at day 3 post-infection (**Figure 2A**), that corresponds to a 3.8-fold decrease. Although this down-regulation was also detected for TWEAK 2 in the kidney after 3 days of infection, in this case, the differences were not significant (**Figure 2A**). No significant changes in TWEAK 1 or 2 mRNA levels were observed in the spleen (**Figure 2B**), gill or liver (data not shown) in response to the virus.

### Regulation of TWEAK 1 and 2 Transcription During PKD

**Figure 3:**
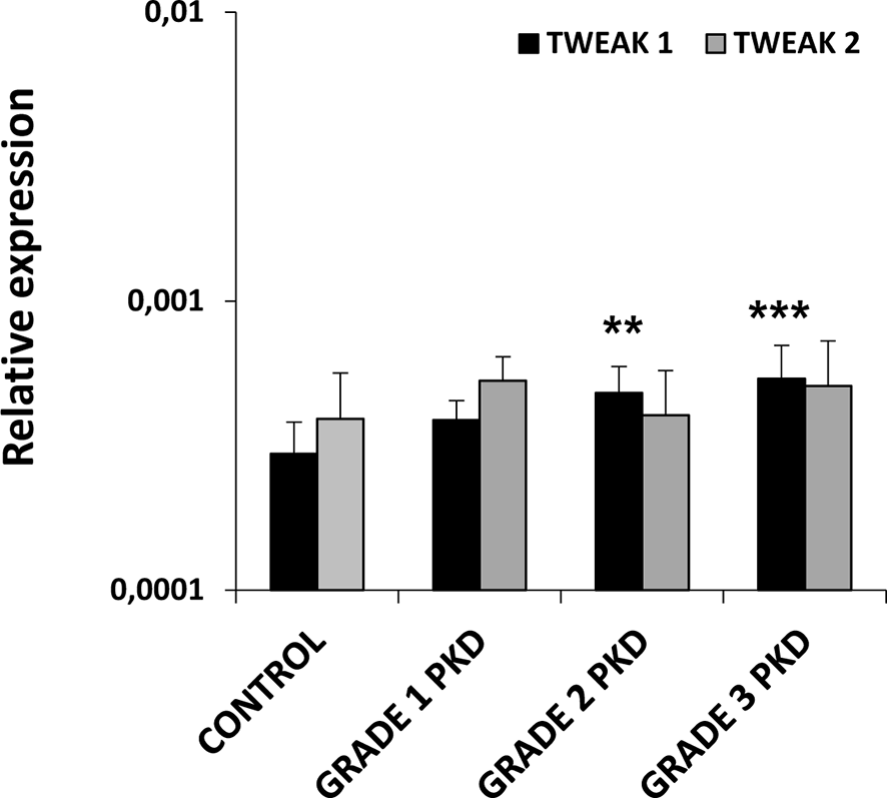
Levels of transcription of TWEAK 1 and 2 in the kidney of rainbow trout naturally infected with the parasite *Tetracapsuloides bryosalmone.* Fish were classified in three different groups attending to the kidney swelling degree (grade 1, 2 and 3) and the levels of TWEAK 1 and 2 determined in the kidney by real-time PCR and compared to those of control fish not exposed to the parasite. Data are shown as the gene expression relative to the expression of an endogenous control EF-1α (mean + SD, n = 4-10). Asterisks denote statistically significant differences between the values obtained in infected fish and those obtained in controls (**p < 0.01, ***p < 0.001).

Given that some reports in rodents have described that TWEAK signaling can contribute to increase the clinical severity of autoimmune/inflammatory diseases such as RA, SLE or MS (7, 8), we decided to study TWEAK transcription in a proliferative/inflammatory condition such as that produced by the parasite *Tetracapsuloides bryosalmonae* during PKD. Our results showed that fish with the highest swelling grades (2 and 3) had a significantly increased TWEAK 1 gene transcription in the kidney when compared to control rainbow trout (**Figure 3**), that corresponded to 1.6 and 1.8-fold increases, respectively. On the contrary, no significant changes were found in TWEAK 2 transcriptional levels between PKD affected and control fish (**Figure 3**).

### Regulation of TWEAK 1 and 2 Transcription in Stimulated HK Leukocytes

**Figure 4:**
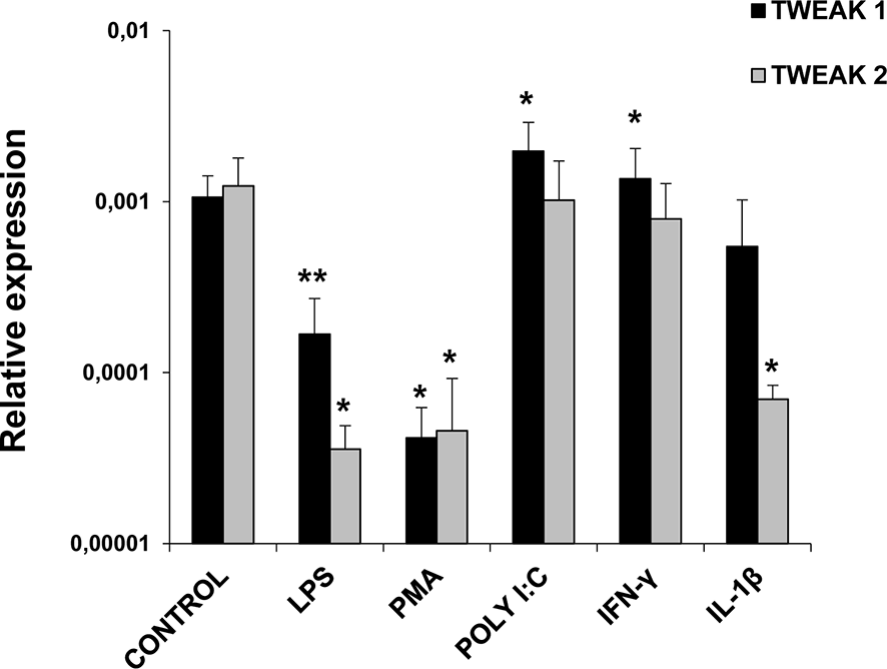
TWEAK 1 and 2 transcription in stimulated HK leukocytes. HK leukocytes were stimulated with LPS (100 µg/ml), PMA (100 ng/ml), Poly I:C (50 µg/ml), IFN-γ (25 ng/ml), IL-1β (20 ng/ml) or were left untreated (unstimulated controls). After 24 h of incubation at 20°C, RNA was extracted and levels of expression of TWEAK 1 and 2 assessed by real-time PCR. Gene expression data was normalized against the endogenous control EF-1α and is shown as relative expression levels (mean + SD; n = 6). Asterisks denote statistically significant differences between the values obtained in stimulated cells and those obtained in unstimulated controls (*p < 0.05, **p < 0.01).

HK isolated leukocytes were treated in the presence or absence of different cell stimuli in order to evaluate the levels of expression of TWEAK 1 and 2 after 24 h of incubation. We observed a significant up-regulation in TWEAK 1 transcriptional levels in HK leukocytes stimulated with poly I:C and rainbow trout IFN-γ in comparison with unstimulated cells (**Figure 4**). In contrast, a significant down-regulation of both TWEAK 1 and 2 transcription was observed when cells were treated with LPS or PMA (**Figure 4**). Finally, TWEAK 2 transcription was also significantly decreased upon treatment with rainbow trout IL-1β (**Figure 4**).

### Transcriptional Changes Induced by TWEAK 1 in HK Leukocytes

**Figure 5:**
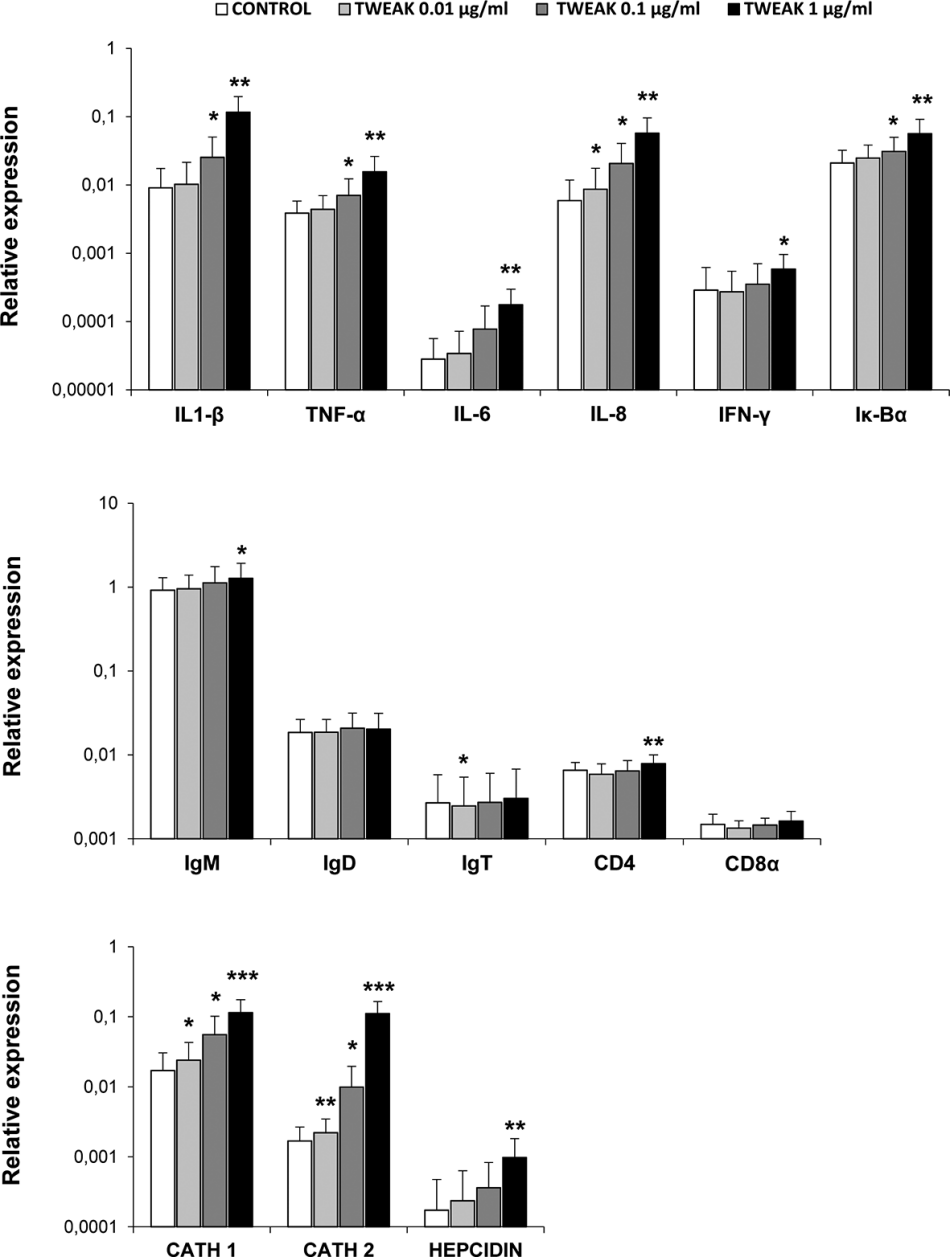
Transcriptional regulation of HK leukocytes in response to TWEAK 1. HK leukocytes (2 x 10^6^ cells/ml) were incubated with different concentrations of recombinant TWEAK 1 (0.01, 0.1 and 1 µg/ml) or left untreated for 24 h. Thereafter, RNA was extracted and the transcription levels of several immune genes [IL-1β, TNF-α, IL-6, IL-8, IFNγ, Iκ-Bα, IgM, IgD, IgT, CD4, CD8a, cathelicidin 1 (CATH 1) and 2 (CATH 2) and hepcidin] evaluated by real-time PCR. Gene expression data were normalized against the endogenous control EF-1α and are shown as relative expression levels (mean + SD; n = 9). Asterisks indicate significantly different values between treated and control cells (*p < 0.05, **p < 0.01, ***p < 0.001).

Next, we assessed the transcriptional effects of TWEAK 1 on HK leukocytes. For this, we stimulated leukocytes with different concentrations of recombinant TWEAK 1 (0.01, 0.1 and 1 µg/ml) and incubated them during 24 h at 20°C. Non-stimulated controls were also included. Subsequently, we evaluated the levels of transcription of a wide range of immune genes, including pro-inflammatory genes (IL-1β, TNF-α, IL-6, IL-8, IFNγ, Iκ-Bα), all fish Igs (IgM, IgD, IgT), T cell markers (CD4, CD8) and antimicrobial peptides (cathelicidin 1 and 2, hepcidin). The results obtained demonstrated that TWEAK 1 induced a significant up-regulation of the levels of transcription of pro-inflammatory genes (IL-1β, TNF-α, IL-6, IL-8 and IFN-γ) as well as in the NF-κB inhibitor Iκ-Bα, known to be up-regulated after NF-κB activation (44) (**Figure 5**). This increase was dose-dependent and reached its highest levels with a dose of 1 µg/ml. Furthermore, IgM and CD4 mRNA levels were slightly but significantly up-regulated when HK leucocytes were treated with 1 µg/ml of recombinant TWEAK 1 compared to those observed in control cells (**Figure 5**). Concerning antimicrobial peptides, a significant dose-dependent increase in the levels of transcription of cathelicidin 1 and 2 and hepcidin was observed in response to TWEAK 1 (**Figure 5**). The levels of gene transcription obtained in cultures exposed to the same volume of TWEAK storage buffer as that used in the TWEAK-treated cultures was similar to that of control untreated cells (data not shown).

To further investigate on which cells within the HK leukocyte cultures was TWEAK 1 exerting its transcriptional effects, we decided to assess the effects of TWEAK 1 on FACS sorted subpopulations from the HK. For this, total leukocytes that had been incubated for 24 h with or without 1 µg/ml of TWEAK 1 were stained with an anti-IgM Ab. IgM^+^ B cells, IgM^-^ lymphoid cells and IgM^-^ myeloid cells were then sorted based on their FSC/SSC profiles and on the fluorescence emitted by the anti-IgM Ab coupled to FITC. The levels of transcription of some of the genes that had been significantly modulated by TWEAK 1 in total HK leukocyte cultures were then assessed in the sorted populations. It has to be taken into account that what we designate as the myeloid population could also contain plasma cells which are also large cells with high complexity. To avoid this, we only sort IgM^-^ myeloid cells as reports in human (45) and rainbow trout (39) have demonstrated that IgM plasma cells retain IgM on the cell membrane.

**Figure 6:**
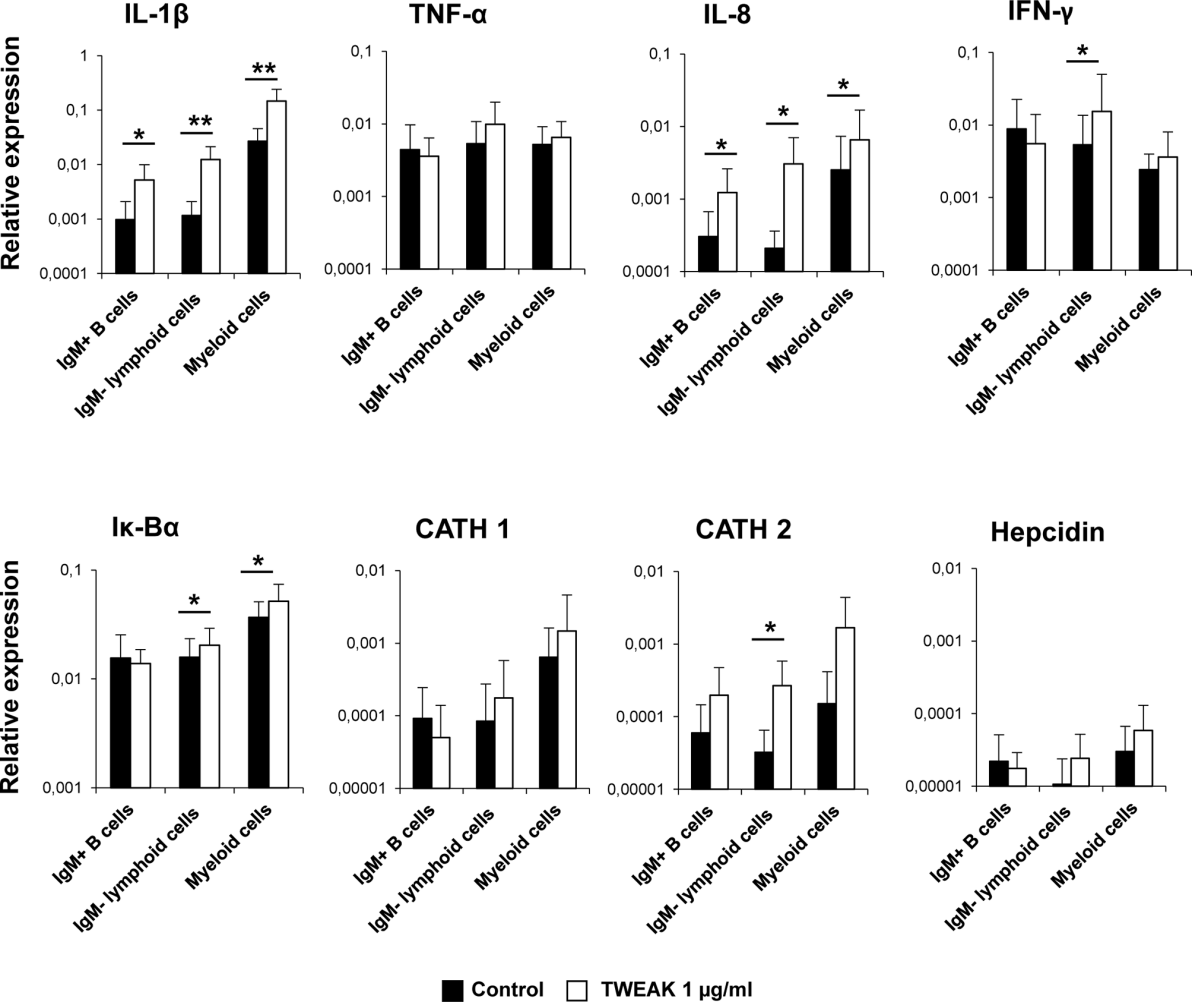
Transcriptional regulation of sorted HK leukocyte populations in response to TWEAK 1. HK leukocytes (2 x 10^6^ cells/ml) were incubated with or without recombinant TWEAK 1 at a concentration of 1 µg/ml. After 24 h of incubation, IgM^+^ B cells, IgM^-^ lymphoid cells and myeloid cells from stimulated and control cultures were FACS sorted attending to the expression of IgM and the FSC/SSC profile. RNA was extracted to determine the levels of transcription of IL-1β, TNF-α, IL-8, IFN-γ, IgM, Iκ-Bα, cathelicidin 1 (CATH 1) and 2 (CATH 2) and hepcidin. Gene expression data were normalized against the endogenous control EF-1α and are shown as relative expression levels (mean + SD; n = 9). Asterisks represent significant differences between control and treated cells (*p ≤ 0.05, **p ≤ 0.01).

Our results revealed that TWEAK 1 had the capacity to increase the transcription of IL-1β and IL-8 in the three sorted subpopulations (**Figure 6**), demonstrating the capacity of this cytokine to regulate inflammation by affecting a wide range of leukocyte subsets. In contrast, only IgM^-^ lymphoid cells and myeloid cells significantly up-regulated Iκ-Bα mRNA levels when incubated with TWEAK 1 (**Figure 6**). In the case of cathelicidin 2 and IFN-γ, it was the IgM^-^ lymphoid subpopulation that seemed to be responding to TWEAK 1, as it was the only subpopulation in which TWEAK 1 provoked significant effects (**Figure 6**). Although TWEAK 1 had shown to up-regulate the transcription of TNF-α, cathelicidin 1 and hepcidin in total HK cultures (**Figure 5**), no significant changes in the levels of transcription of these genes were observed when mRNA levels were studied in sorted subpopulations (**Figure 6**).

### Effects of TWEAK 1 on IgM^+^ B Cell Functions

**Figure 7:**
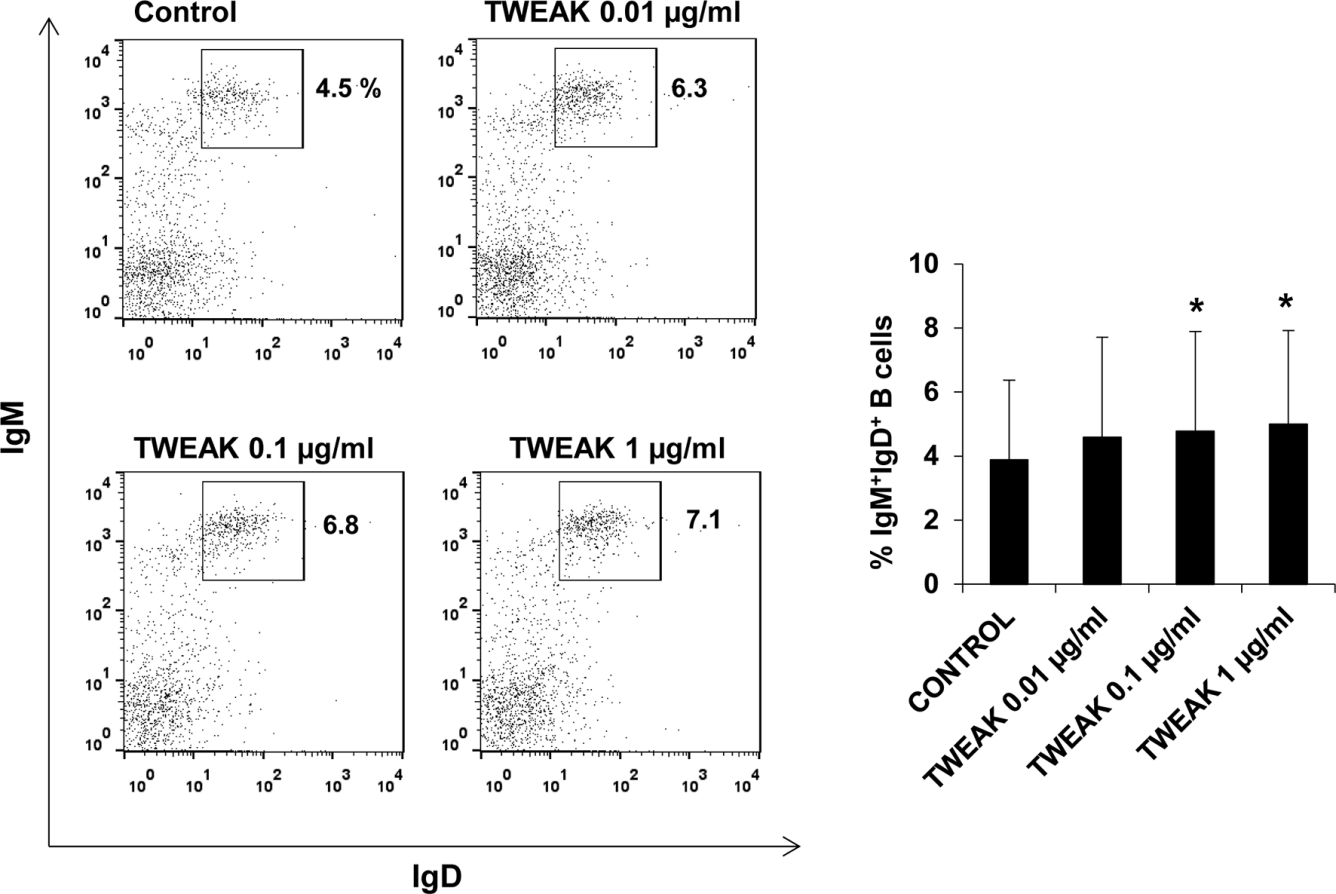
Effect of TWEAK 1 on the survival of HK IgM^+^IgD^+^ B cells. HK leukocytes were incubated with recombinant TWEAK 1 at concentrations of 0.01, 0.1 and 1 µg/ml or with media alone (control) during 3 days at 20°C. The percentage of IgM^+^IgD^+^ B cells was then determined by flow cytometry using specific mAbs. Representative dot plots (left) are included along with a graph showing mean + SD percentages of IgM^+^IgD^+^ B cells obtained in 9 independent fish (right). Asterisks denote significant differences between control and treated cells (*p ≤ 0.05).

Having established that TWEAK 1 regulated IL-1β and IL-8 transcription in HK IgM^+^ B cells, we decided to study whether the cytokine could affect other functions of B cells. First, we determined the survival of IgM^+^IgD^+^ B cells in HK leukocyte cultures treated with different rTWEAK concentrations (0.01, 0.1 and 1 µg/ml) or with media alone. Our results demonstrated that after 72 h the percentage of IgM^+^IgD^+^ B cells in HK cultures was significantly increased in cultures treated with 0.1 and 1 µg/ml of TWEAK 1 compared to the percentages obtained in unstimulated cultures (**Figure 7**). This increased survival of IgM^+^IgD^+^ B cells was accompanied by a subtle but significant increase in the levels of IgM expressed in the cell membrane (data not shown).

**Figure 8:**
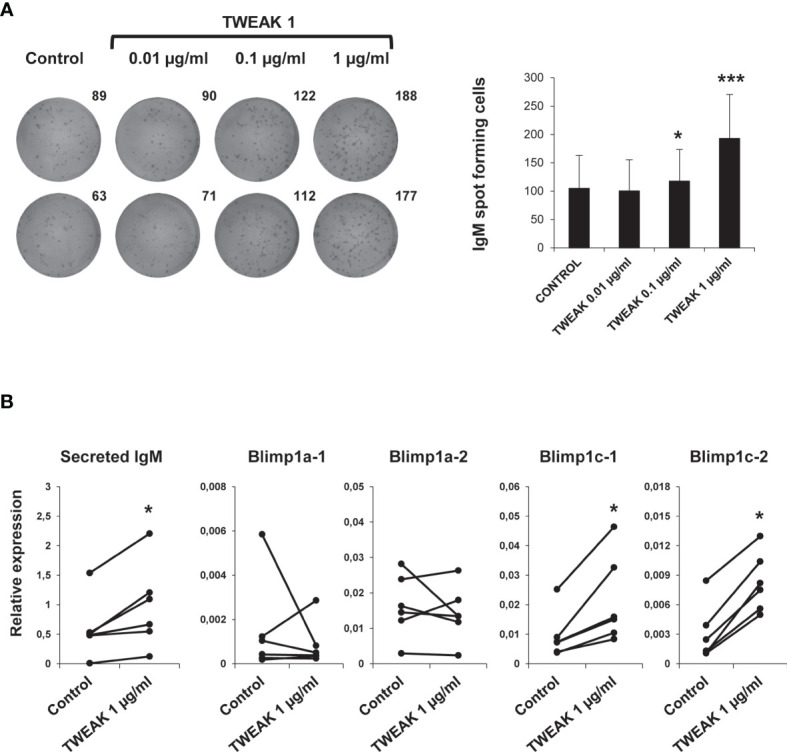
Effect of TWEAK 1 on the differentiation of HK IgM^+^ B cells. HK leukocytes were cultured with different doses of recombinant TWEAK 1 (0.01, 0.1 and 1 µg/ml) or with media alone for 48 h and then plated in ELISpot plates coated with anti-IgM mAb for further 24 h. Next, cells were washed away and a biotinylated anti-IgM used to evaluate the number of spot forming cells. **(A)** Duplicate wells from a representative experiment are shown (left) along with the mean number of spot forming cells per well (right) (mean + SD; n = 9). **(B)** The levels of transcription of secreted IgM, Blimp1a-1, Blimp1a-2, Blimp1c-1, and Blimp1c-2 were assessed in sorted IgM^+^ B cells obtained from HK leukocyte cultures stimulated with 1 µg/ml TWEAK 1 or from control cultures by real-time PCR. Results are shown as the gene expression relative to the expression of an endogenous control EF-1α for each individual fish (n = 6). Asterisks denote significant differences between stimulated and control cells (*p < 0.05; ***p < 0.001).

To assess if TWEAK 1 had the capacity to induce the differentiation of HK IgM^+^ B cells, we studied the number of IgM secreting cells in HK leukocyte cultures after stimulation with different TWEAK doses. The ELISpot results obtained revealed a significant increase in the number of IgM secreting cells in cultures treated with TWEAK 1 at 0.1 and 1 µg/ml in comparison to untreated cells (**Figure 8A**). This increase was not observed when HK leukocytes were incubated with an irrelevant protein produced in the same conditions (**Supplementary Figure S5**) or when exposed to the same volume of TWEAK storage buffer than that used in TWEAK-treated cultures (data not shown). Accordingly, a significant up-regulation in the levels of transcription of secreted IgM and two homologues of mammalian Blimp1 (Blimp1c-1 and Blimp1c-2) was observed in IgM^+^ B cells stimulated with 1 µg/ml TWEAK 1 in comparison with untreated cells (**Figure 8B**). Given that Blimp1 is an essential transcription factor required for the development of plasma cells, the results obtained point to the capacity of TWEAK to induce B cell differentiation in teleost fish.

## Discussion

TWEAK is an important member of the TNF superfamily that exerts a wide range of immune actions and seems to contribute to the pathogenesis of several diseases. Although some studies in mammals have aimed at characterizing TWEAK at both molecular and functional levels (3, 9–11), there are still many aspects of its regulation and function that are not yet known. The information available is even scarcer in teleost fish, in which TWEAK homologues have only been identified in a limited number of species (25–28).

Mammalian TWEAK has been shown to be involved in different pathological conditions including tumor growth, chronic autoimmune diseases or acute ischemic stroke (5, 7, 8). Nevertheless, even in mammals, only a few studies have evaluated the role of TWEAK in infectious diseases. Human TWEAK expression was shown to increase in early mycobacterial infections, inducing autophagy and mycobacterial autophagosome maturation (46). Interestingly, TWEAK serum levels were increased in SARS-CoV-2 patients in comparison with healthy individuals (47), whereas human papillomavirus 16 (HPV16) infection increased TWEAK expression in keratinocytes from infected tissues (48). In goat, TWEAK production was increased in response to Peste des Petits Rumiants virus (PPRV), where it modulated NK cell function during the infection (49). In grass carp, TWEAK transcription was significantly up-regulated after *Aeromonas hydrophila* in the liver, gill, kidney and spleen (27). When grass carp were infected with *Aquareovirus*, the highest increases in TWEAK transcription were observed in skin and gills (27). In contrast, when rainbow trout were infected with VHSV, we observed a significant decrease of TWEAK 1 transcription levels in the kidney at day 3 post-challenge. Furthermore, no transcriptional changes were observed in spleen, gills (data not shown) or liver (data not shown) in response to the virus. Of course, these different responses could be due to the different regulation of TWEAK 1 transcription in different teleost species. Alternatively, it could be a consequence of the fact that each viral agent and especially those from different families, provoke a specific pathogenesis, and replicate and affect a range of tissues and cell types that could be very different. Additionally, we cannot rule out the possibility that some specific viruses have the capacity to inhibit the transcription of some cytokines such as TWEAK during the infection process to evade the immune response or that immune cells expressing TWEAK migrate to other peripheral immune organs not analyzed in this work. Thus, further studies are required to understand the role of TWEAK in viral infections in fish. On the other hand, PKD is an immune-pathological condition characterized by an exacerbated host lymphocyte response to *T*. *bryosalmonae* that induces a chronic kidney pathology (35, 50, 51). In this case, we did observe a significant increase in the levels of transcription of TWEAK 1 in the most severe grades of the disease (grades 2 and 3). It is noteworthy mentioning that other members of TNFSF such as BAFF and APRIL were also significantly up-regulated during PKD (52). In particular, BAFF was significantly up-regulated in grade 3 whereas APRIL was significantly up-regulated in all disease grades in comparison with healthy fish. Interestingly, the levels of transcription of BAFF and APRIL correlated with total IgM mRNA levels, known to be highly augmented during the course of PKD (52). Additionally, BAFF transcription correlated with secreted IgT mRNA levels (52). These results suggested that BAFF and APRIL were at least in part responsible for this increased Ig production. Thus, knowing now that TWEAK 1 is also induced during PKD and that it promotes IgM secretion in the HK, it would be reasonable to think that TWEAK could also be, at least partially, mediating the hypergammaglobulemia provoked by the parasite in this tissue.

We further investigated how TWEAK transcription was modulated in the HK by incubating HK leukocytes *in vitro* with different immune stimuli. In our model, LPS and PMA-treated HK leukocytes down-regulated both isoforms of TWEAK after 24 h in comparison with unstimulated controls. Similarly, mice treated with LPS *in vivo* underwent a rapid disappearance of TWEAK mRNA in all tested tissues (kidney, heart, spleen and liver) (14). Moreover, LPS treatment of murine peritoneal macrophages treated with thioglycolate showed a rapid decrease in the levels of TWEAK transcription. In these cases, the loss of TWEAK mRNA in mouse tissues and macrophages treated with LPS was hypothesized to be associated to its rapid destabilization or to a higher turnover because of a rapid increase of its translation. Further studies are necessary to clarify whether this is the reason for the down-regulations in TWEAK mRNAs observed in rainbow trout in response to VHSV or to stimuli such as LPS, PMA or IL-1β (in the case of TWEAK 2). In human, TWEAK cell surface expression was quickly induced on blood monocytes after IFN-γ stimulation (11). Likewise, rainbow trout TWEAK 1 mRNA showed a significant up-regulation in HK leukocytes after exposure to recombinant IFN-γ. Poly I:C also significantly up-regulated TWEAK 1 transcription, again pointing to a role of TWEAK in antiviral responses in fish.

In mammals, TWEAK binding to Fn14 has been shown to stimulate the release of cytokines such as TNF-α, IL-1, IL-6, G-CSF, IFN-γ, MCP1 (monocyte chemoattractant protein 1), MIP1-α (macrophage inflammatory protein 1-α), VCAM-1 (vascular adhesion molecule 1) or IP-10 (IFNγ-induced protein 10) (53–55). Hence, the TWEAK/Fn14 signaling pathway contributes to promote inflammation in different tissues, reason for which the exacerbated or persistent upregulation of this pathway has been demonstrated to play a crucial role in the pathogenesis of some autoimmune/inflammatory diseases such as SLE and RA (6, 56, 57). In our experiments, TWEAK 1 induced the transcription of several pro-inflammatory genes such as IL-1β, TNF-α, IL-6, IL-8, and IFN-γ in rainbow trout HK leukocytes. When we studied the expression of these pro-inflammatory cytokines in sorted HK leukocyte subpopulations we found a significant increase in the mRNA levels of IL-1β and IL-8 in the three analyzed subpopulations; this is, IgM^+^ B cells, IgM^-^ lymphoid cells and IgM^-^ myeloid cells. These results demonstrate that, in fish, TWEAK has the capacity to induce the production of pro-inflammatory cytokines in a wide range of leukocyte types. Additionally, TWEAK also promoted the transcription of several antimicrobial peptides in HK leukocytes. Although our results using sorted leukocyte populations do not clearly indicate which cells are up-regulating these proteins in response to TWEAK, to our knowledge, our study constitutes the first report linking TWEAK with the production of antimicrobial peptides. It is important to mention, that the rainbow trout TWEAK 1 sequence contains several potential furin cleavage sites. Whether some or all of these sites are active *in vivo* is something that should be explored in future studies, as the bioactivity of the protein might differ if these alternative cleavage sites are utilized.

In mammals, TWEAK-Fn14 signaling induces the interaction with TRAF (TNF receptor-associated factor) proteins, activating both the canonical and the non-canonical NF-κB signaling pathway (6). The canonical NF-κB pathway is characterized by phosphorylation of IκB and translocation of p50/p65 heterodimers into the nucleus, activating different cellular functions such as inflammation, cell survival/proliferation, cell death or cell migration (6). Accordingly, we found that Iκ-Bα was significantly up-regulated in TWEAK-stimulated leukocytes, pointing to an activation of the canonical NF-κB pathway during the cellular response to TWEAK in fish. Interestingly, when we analyzed Iκ-Bα transcription in individual HK leukocyte subpopulations we found significant up-regulation in IgM^-^ lymphoid cells and myeloid cells, but not in IgM^+^ B cells, suggesting differential effects of TWEAK in the activation of NF-κB in diverse leukocyte subpopulations.

Although a few studies have established an effect of TWEAK on B cell immune responses in mammals (23, 24), the exact mechanism through which TWEAK modulates B cells is still not fully understood. In the current study, we first demonstrated that HK IgM^+^ B cells have the capacity to transcribe both TWEAK orthologues. Similarly, sorted rainbow trout IgM^+^ B cells obtained from spleen had been shown to constitutively transcribe TWEAK 1, transcription that was significantly down-regulated in response to TNP-LPS (29). In RA, sub-populations of CD22^+^ B cells and CD38^+^ plasma cells in synovial samples that seemed to be involved in the RA pathogenesis were shown to express TWEAK (58). Similarly, TWEAK expression has also been shown in plasma cells from patients with multiple myeloma (59).

Additionally, we demonstrate the involvement of TWEAK in regulation and differentiation of teleost B cells, as TWEAK 1 increased both IgM^+^ B cell survival and the number of IgM secreting cells in HK leukocytes cultures. Of course, the increased IgM secretion could be a consequence of the higher survival of IgM^+^ B cells in cultures. However, our results showing that recombinant TWEAK 1 up-regulated the transcription of secreted IgM and two homologues of Blimp1 (Blimp1-c1 and c2) in sorted IgM^+^ B cells points to a clear role of TWEAK 1 in trout B cell differentiation. Of course, additional studies would be needed to unequivocally establish that these actions on B cells are direct TWEAK actions. However, the fact that rainbow trout IgM^+^ B cells are known to express the two homologues of the TWEAK receptor (29) strongly suggests that the effects exerted by TWEAK on trout B cells are direct effects. Interestingly, previous results from our group revealed that these two homologues of mammalian Fn14 were transcriptionally up-regulated in response to stimulation with either TNP-LPS or TNP-KLH (29).

In summary, in the current study we have undertaken, to our knowledge, the first functional characterization of teleost TWEAK. After having established that salmonids contain two genes that code for different TWEAK homologs, possibly as a result of the specific whole genome duplication event that these species suffered through evolution (60), we studied the regulation of transcription of both genes in response to different pathogenic exposures. The fact that TWEAK 1 was modulated in response to VHSV and that TWEAK 1 transcription was induced by poly I:C in HK leukocytes point to an important role of this cytokine in the teleost antiviral response. Furthermore, the fact that TWEAK 1 transcription was induced during PKD and knowing that TWEAK 1 is a survival and differentiation factor for rainbow trout B cells, suggest a role for TWEAK in the B cell dysregulatory effects provoked by *T. bryosalmonae.* Finally, we have established that, in teleost, TWEAK induced not only the transcription of pro-inflammatory genes but also that of antimicrobial peptides. Our results demonstrate that rainbow trout TWEAK is a pleiotropic cytokine with differential effects on many leukocyte subsets and provide interesting information regarding the regulation and function of this yet understudied cytokine.

## Data Availability Statement

The raw data supporting the conclusions of this article will be made available by the authors, without undue reservation.

## Ethics Statement

The animal study was reviewed and approved by Ethics Committee from INIA (Code PROEX 002/17).

## Author Contributions

BA performed and analyzed all the experiments with help from EP-F. EM provided support with flow cytometry analyses and performed the cell sortings. PP performed sequence similarity searches and designed and performed all phylogenetic analyses. CT conceived the work, designed the experiments and wrote the final version of the manuscript with help from BA. All authors contributed to the article and approved the submitted version.

## Funding

This work was supported by the European Research Council (ERC Consolidator Grant 2016 725061 TEMUBLYM) and by the Spanish Ministry of Science, Innovation and Universities (project AGL2017-85494-C2-1-R).

## Conflict of Interest

The authors declare that the research was conducted in the absence of any commercial or financial relationships that could be construed as a potential conflict of interest.

## Publisher’s Note

All claims expressed in this article are solely those of the authors and do not necessarily represent those of their affiliated organizations, or those of the publisher, the editors and the reviewers. Any product that may be evaluated in this article, or claim that may be made by its manufacturer, is not guaranteed or endorsed by the publisher.
