# Supplementary material for: Pro-Inflammatory and B Cell Regulating Capacities of TWEAK in Rainbow Trout (Oncorhynchus mykiss)
Source: Front Immunol. 2021 Oct 1;12:748836. doi: 10.3389/fimmu.2021.748836 (PMC8517431; doi:10.3389/fimmu.2021.748836)
Supplement: Supplementary file 1 [file Presentation_1.pptx]

## Slide 1
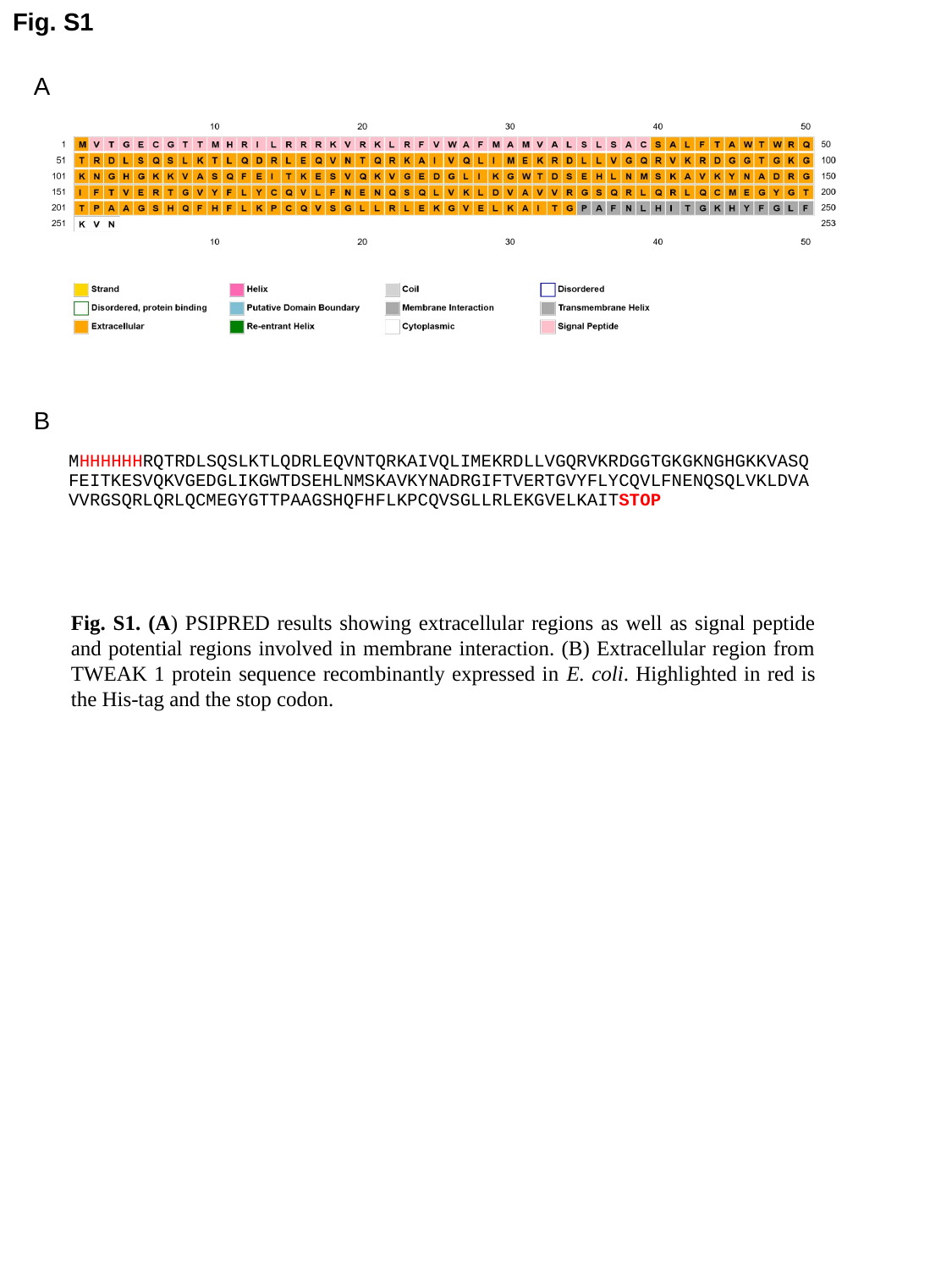

Fig. S1
A
B
Fig. S1. (A) PSIPRED results showing extracellular regions as well as signal peptide and potential regions involved in membrane interaction. (B) Extracellular region from TWEAK 1 protein sequence recombinantly expressed in E. coli. Highlighted in red is the His-tag and the stop codon.

## Slide 2
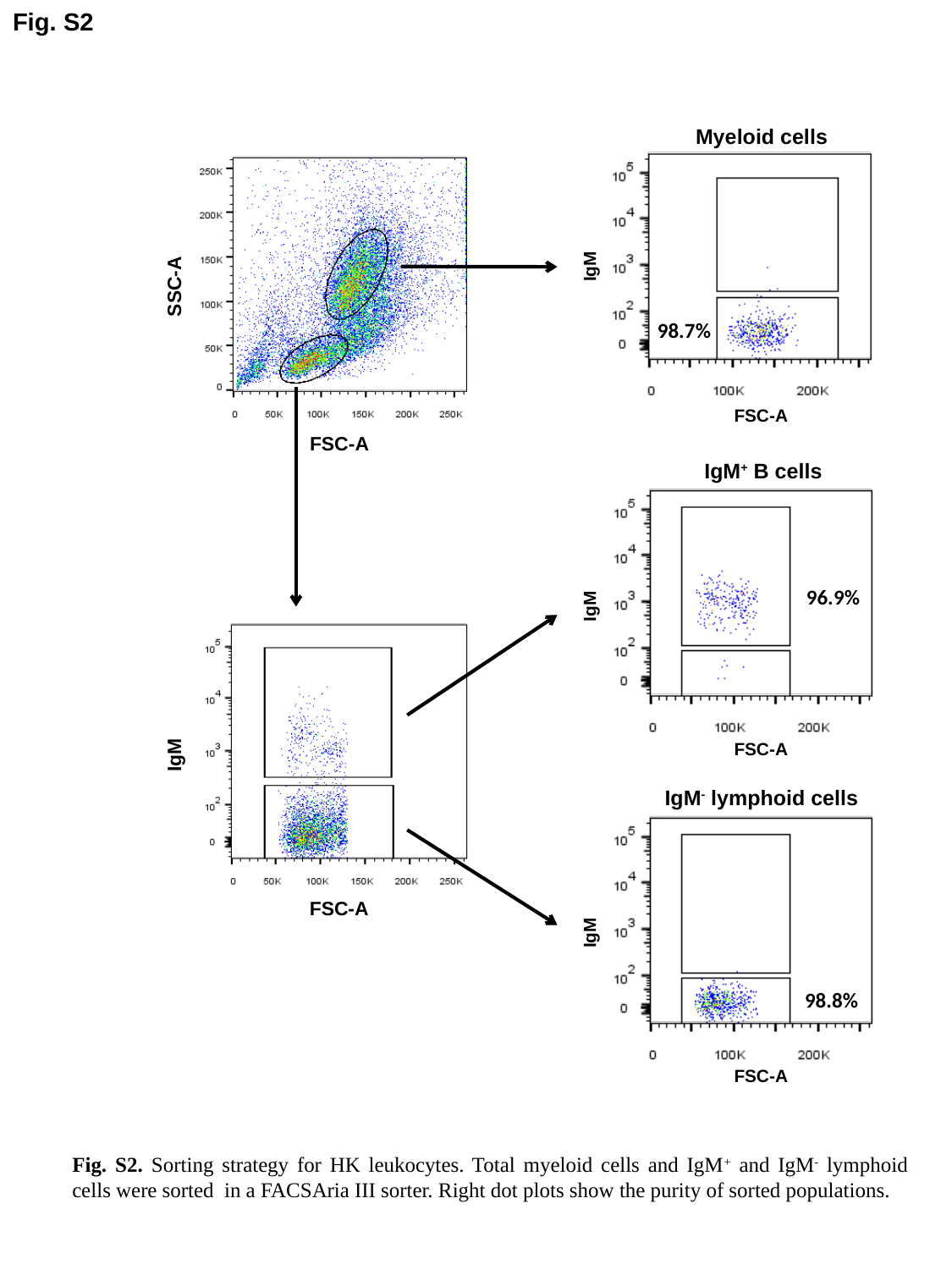

Fig. S2
Myeloid cells
IgM
SSC-A
98.7%
FSC-A
FSC-A
IgM+ B cells
96.9%
IgM
FSC-A
IgM
IgM- lymphoid cells
FSC-A
IgM
98.8%
FSC-A
Fig. S2. Sorting strategy for HK leukocytes. Total myeloid cells and IgM+ and IgM- lymphoid cells were sorted in a FACSAria III sorter. Right dot plots show the purity of sorted populations.

## Slide 3
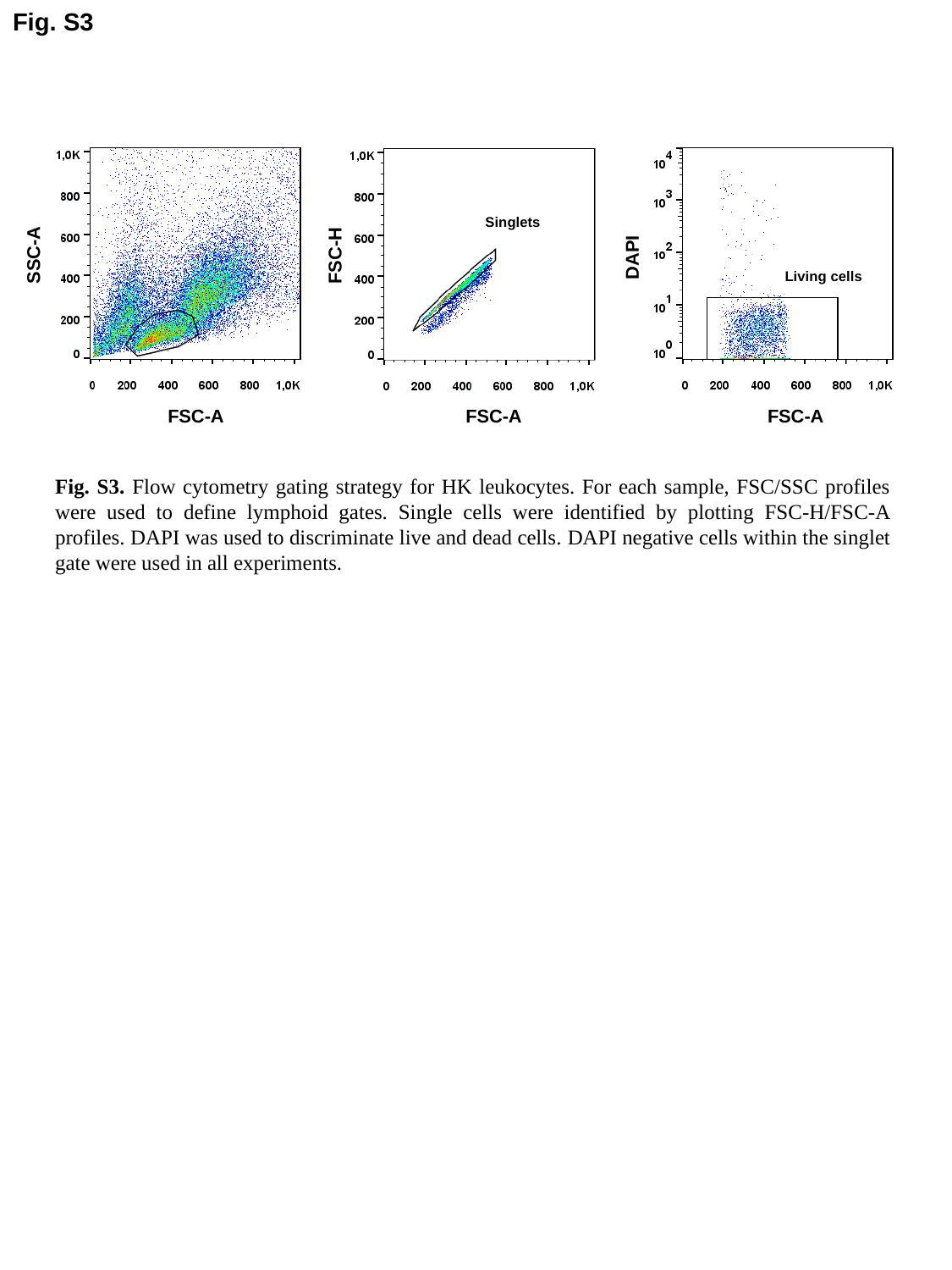

Fig. S3
Singlets
DAPI
FSC-H
SSC-A
Living cells
FSC-A
FSC-A
FSC-A
Fig. S3. Flow cytometry gating strategy for HK leukocytes. For each sample, FSC/SSC profiles were used to define lymphoid gates. Single cells were identified by plotting FSC-H/FSC-A profiles. DAPI was used to discriminate live and dead cells. DAPI negative cells within the singlet gate were used in all experiments.

## Slide 4
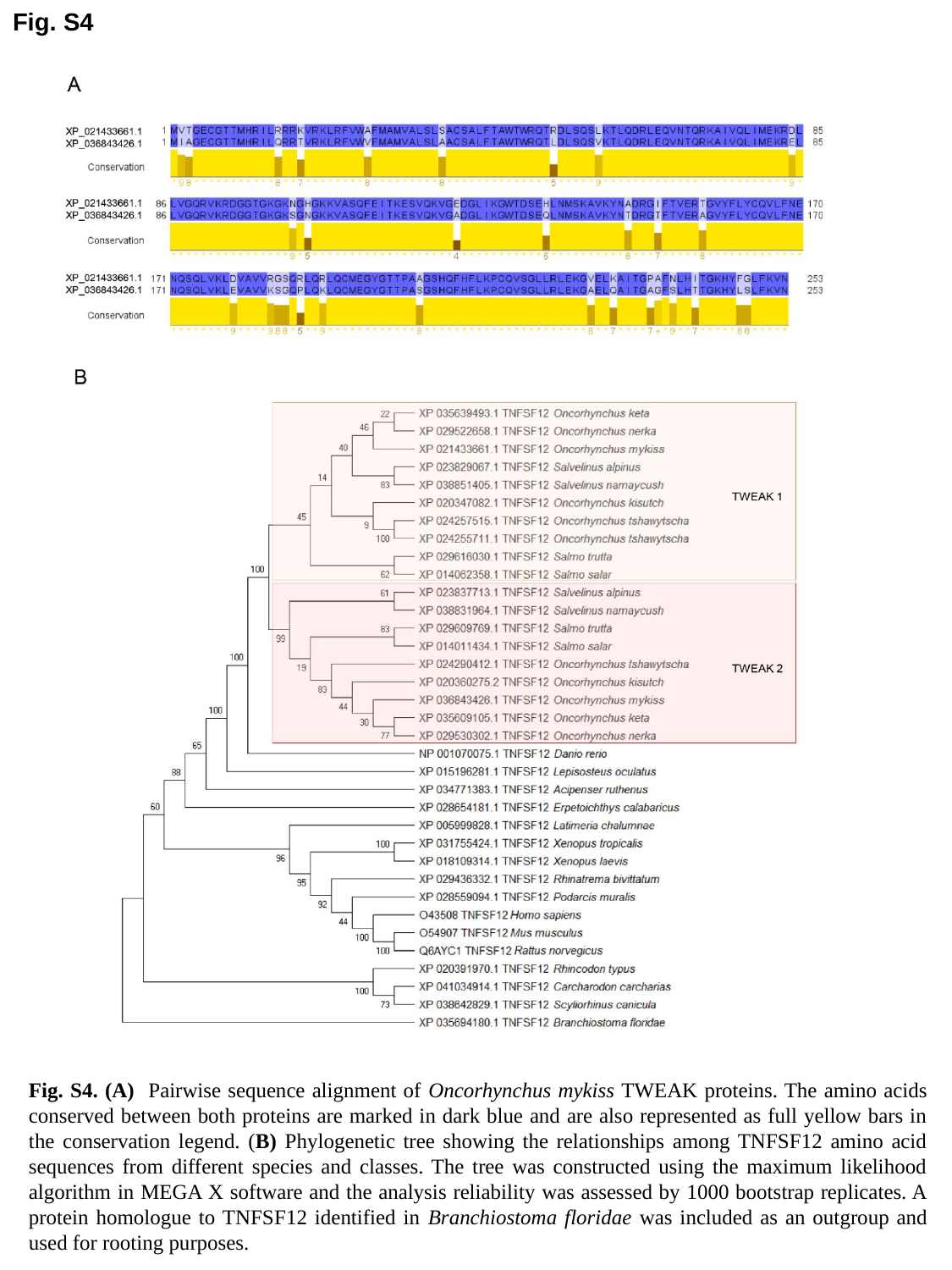

Fig. S4
Fig. S4. (A) Pairwise sequence alignment of Oncorhynchus mykiss TWEAK proteins. The amino acids conserved between both proteins are marked in dark blue and are also represented as full yellow bars in the conservation legend. (B) Phylogenetic tree showing the relationships among TNFSF12 amino acid sequences from different species and classes. The tree was constructed using the maximum likelihood algorithm in MEGA X software and the analysis reliability was assessed by 1000 bootstrap replicates. A protein homologue to TNFSF12 identified in Branchiostoma floridae was included as an outgroup and used for rooting purposes.

## Slide 5
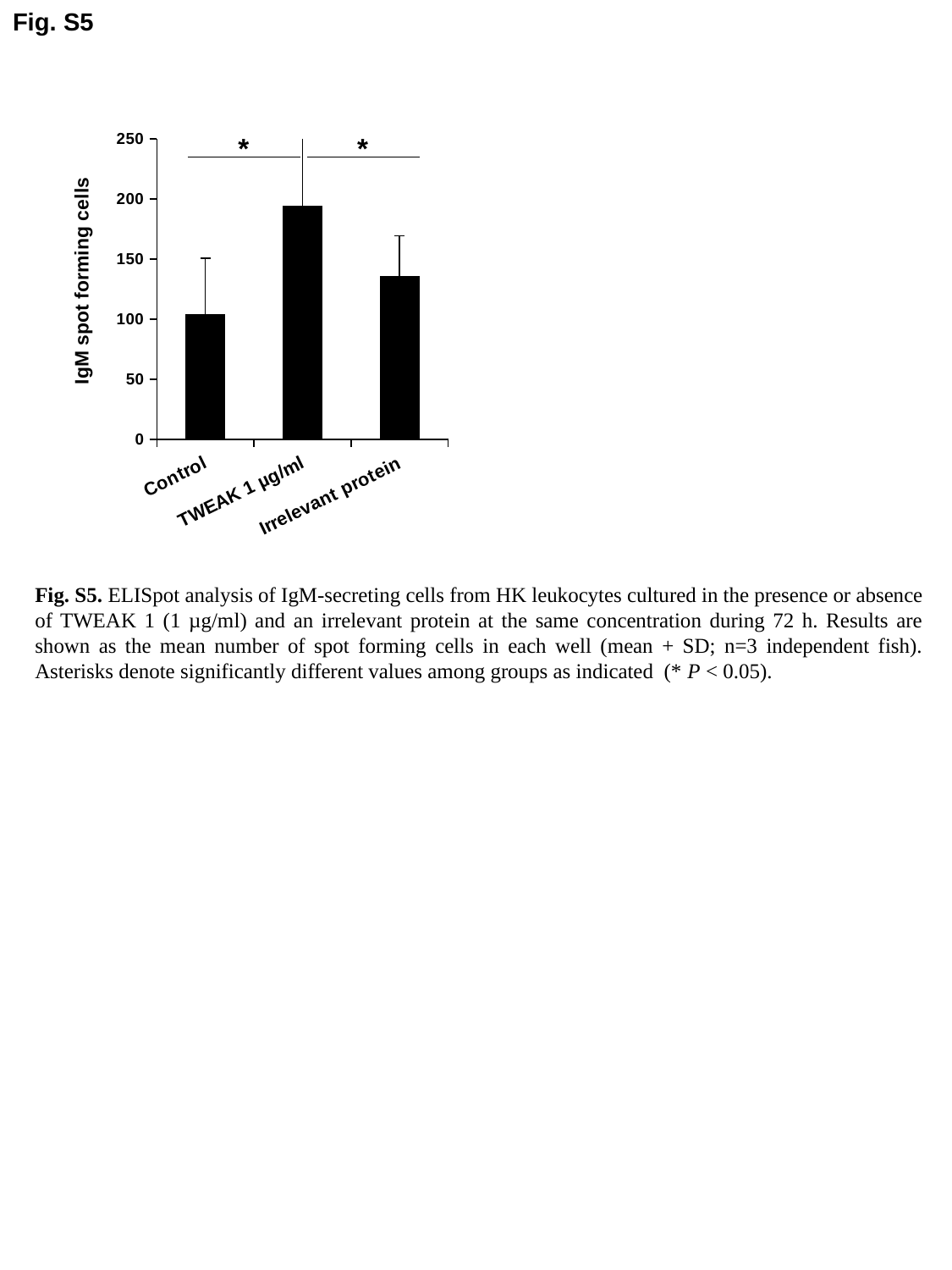

Fig. S5
### Chart
| Category | |
|---|---|
| Control | 103.77777777777777 |
| TWEAK 1 µg/ml | 194.2222222222222 |
| Irrelevant protein | 135.33333333333331 |*
*
IgM spot forming cells
Fig. S5. ELISpot analysis of IgM-secreting cells from HK leukocytes cultured in the presence or absence of TWEAK 1 (1 µg/ml) and an irrelevant protein at the same concentration during 72 h. Results are shown as the mean number of spot forming cells in each well (mean + SD; n=3 independent fish). Asterisks denote significantly different values among groups as indicated (* P < 0.05).

## Slide 6
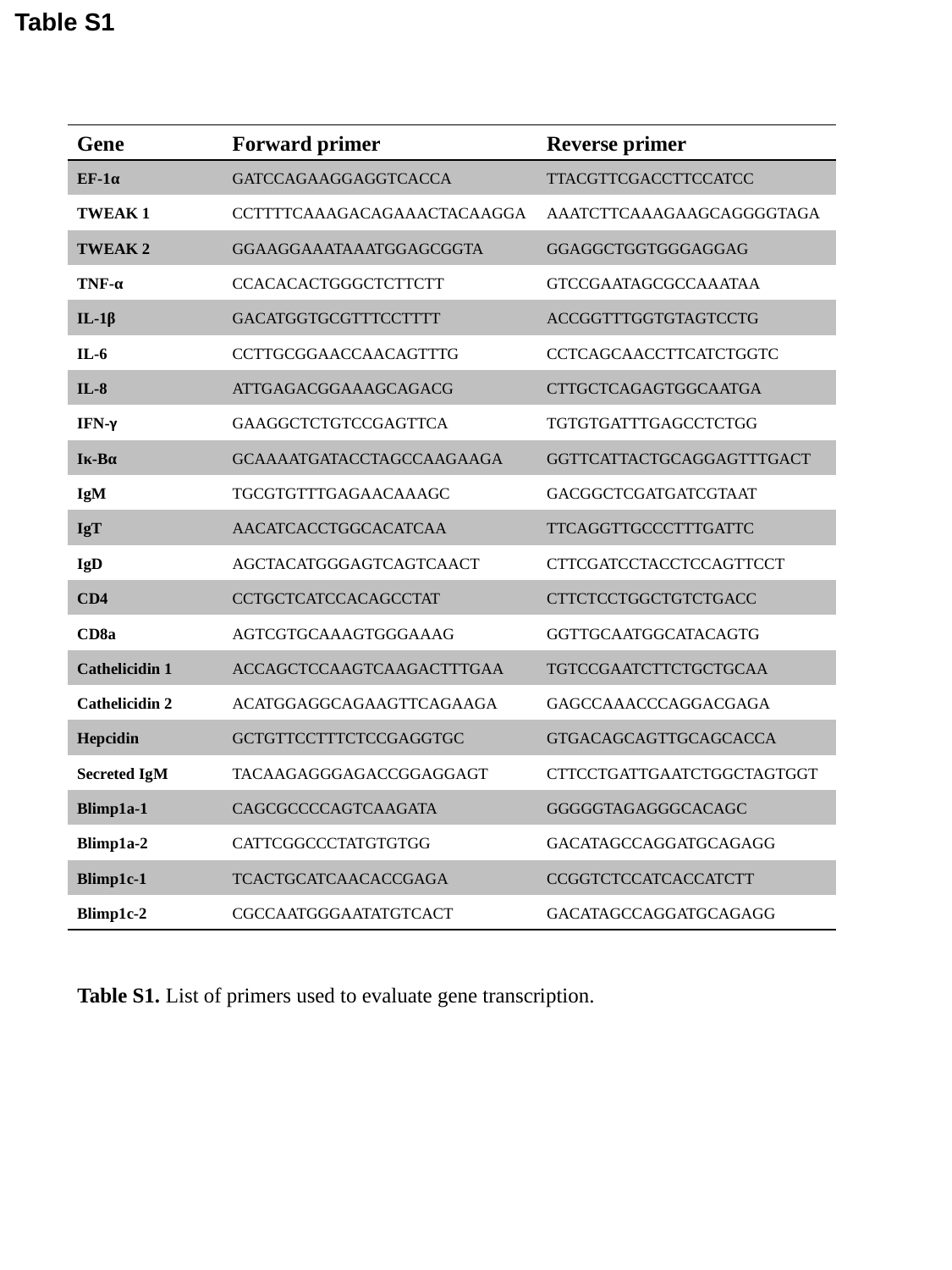

Table S1
| Gene | Forward primer | Reverse primer |
| --- | --- | --- |
| EF-1α | GATCCAGAAGGAGGTCACCA | TTACGTTCGACCTTCCATCC |
| TWEAK 1 | CCTTTTCAAAGACAGAAACTACAAGGA | AAATCTTCAAAGAAGCAGGGGTAGA |
| TWEAK 2 | GGAAGGAAATAAATGGAGCGGTA | GGAGGCTGGTGGGAGGAG |
| TNF-α | CCACACACTGGGCTCTTCTT | GTCCGAATAGCGCCAAATAA |
| IL-1β | GACATGGTGCGTTTCCTTTT | ACCGGTTTGGTGTAGTCCTG |
| IL-6 | CCTTGCGGAACCAACAGTTTG | CCTCAGCAACCTTCATCTGGTC |
| IL-8 | ATTGAGACGGAAAGCAGACG | CTTGCTCAGAGTGGCAATGA |
| IFN-γ | GAAGGCTCTGTCCGAGTTCA | TGTGTGATTTGAGCCTCTGG |
| Iκ-Bα | GCAAAATGATACCTAGCCAAGAAGA | GGTTCATTACTGCAGGAGTTTGACT |
| IgM | TGCGTGTTTGAGAACAAAGC | GACGGCTCGATGATCGTAAT |
| IgT | AACATCACCTGGCACATCAA | TTCAGGTTGCCCTTTGATTC |
| IgD | AGCTACATGGGAGTCAGTCAACT | CTTCGATCCTACCTCCAGTTCCT |
| CD4 | CCTGCTCATCCACAGCCTAT | CTTCTCCTGGCTGTCTGACC |
| CD8a | AGTCGTGCAAAGTGGGAAAG | GGTTGCAATGGCATACAGTG |
| Cathelicidin 1 | ACCAGCTCCAAGTCAAGACTTTGAA | TGTCCGAATCTTCTGCTGCAA |
| Cathelicidin 2 | ACATGGAGGCAGAAGTTCAGAAGA | GAGCCAAACCCAGGACGAGA |
| Hepcidin | GCTGTTCCTTTCTCCGAGGTGC | GTGACAGCAGTTGCAGCACCA |
| Secreted IgM | TACAAGAGGGAGACCGGAGGAGT | CTTCCTGATTGAATCTGGCTAGTGGT |
| Blimp1a-1 | CAGCGCCCCAGTCAAGATA | GGGGGTAGAGGGCACAGC |
| Blimp1a-2 | CATTCGGCCCTATGTGTGG | GACATAGCCAGGATGCAGAGG |
| Blimp1c-1 | TCACTGCATCAACACCGAGA | CCGGTCTCCATCACCATCTT |
| Blimp1c-2 | CGCCAATGGGAATATGTCACT | GACATAGCCAGGATGCAGAGG |
Table S1. List of primers used to evaluate gene transcription.
